# 

**Published:** 1886-01-20

**Authors:** 


VOL. XVI. JAN'Y 20, 1886. NO. 1.

THE

### SOUTHERN MEDICAL RECORD.

LIBRARY INDEXED S.G.O.
9

EDITORS:

T. S. POWELL, M. D. R. C. WORD, M. D.

COLLABORATORS:

J. McF. Gaston, M. D.,
W. D. Bizzell, M. D.,
W. P. Nicolson, M. D.,
E. Van Goidtsnoven, M. D.,
G. G. Roy, M. D.,
A. G. Hobbs, M. D.,
W. S. Elkin, M. D.,
W. A. Crow, M. D.

**Terms: $2.00 per Annum, in Advance; 4 Copies $6.00; Single Copies 20 Cents.**

Address R. C. WORD, M.D., Managing Editor, Atlanta, Ga.

Published and Entered as Second-class Matter at Atlanta, Ga.

